# Distinct urine metabolome after Asian ginseng and American ginseng intervention based on GC-MS metabolomics approach

**DOI:** 10.1038/srep39045

**Published:** 2016-12-19

**Authors:** Liu Yang, Qing-Tao Yu, Ya-Zhong Ge, Wen-Song Zhang, Yong Fan, Chung-Wah Ma, Qun Liu, Lian-Wen Qi

**Affiliations:** 1 State Key Laboratory of Natural Medicines, China Pharmaceutical University, Nanjing, Jiangsu, 210009, P. R. China; 2 Research & Development Centre, Infinitus (China) Company Ltd, Guangzhou 510663, China

## Abstract

Ginseng occupies a prominent position in the list of best-selling natural products worldwide. Asian ginseng (*Panax ginseng*) and American ginseng (*Panax quinquefolius*) show different properties and medicinal applications in pharmacology, even though the main active constituents of them are both thought to be ginsenosides. Metabolomics is a promising method to profile entire endogenous metabolites and monitor their fluctuations related to exogenous stimulus. Herein, an untargeted metabolomics approach was applied to study the overall urine metabolic differences between Asian ginseng and American ginseng in mice. Metabolomics analyses were performed using gas chromatography-mass spectrometry (GC-MS) together with multivariate statistical data analysis. A total of 21 metabolites related to D-glutamine and D-glutamate metabolism, glutathione metabolism, TCA cycle and glyoxylate and dicarboxylate metabolism, differed significantly under the Asian ginseng treatment; 34 metabolites mainly associated with glyoxylate and dicarboxylate metabolism, TCA cycle and taurine and hypotaurine metabolism, were significantly altered after American ginseng treatment. Urinary metabolomics reveal that Asian ginseng and American ginseng can benefit organism physiological and biological functions via regulating multiple metabolic pathways. The important pathways identified from Asian ginseng and American ginseng can also help to explore new therapeutic effects or action targets so as to broad application of these two ginsengs.

Asian ginseng, the dried root and rhizome of *Panax ginseng* C. A. Meyer, is known to reinforce “qi”, invigorating spleen for benefiting lung and tranquilizing the mind and promoting the intelligence. American ginseng is derived from the dried root of *Panax quinquefolium* L., with the property of cool and mainly used to tonify “qi” and nourish “yin”, which means removing “heat” and promoting the production of body fluids^1^. Ginseng saponins are usually considered to be the major bioactive components in these two ginseng species, however, there are somewhat different in their effects. Asian ginseng has a high ratio of Rg1: Rb1 thought to have stimulatory effects on central nervous system (CNS) while American ginseng has a lower ratio of Rg1: Rb1 but can calm the CNS^2^,^3^. The previous literatures evidenced that Asian ginseng and American ginseng exert lowering and neutral effects on blood pressure, respectively^4^,^5^,^6^,^7^. PARK *et al*. concluded that *Panax ginseng* did not change body temperature but a high dose of *Panax quinquefolius* may reduce mice night-time body temperature and pyrogen-related factors^8^. Besides, there are evidences to support that both Asian ginseng and American ginseng can improve human cognitive function^9^,^10^. In addition, several controlled clinical trials demonstrated ginseng’s therapeutic potential for glycemic control whereas Asian ginseng yields greater fast plasma insulin reductions relative to American ginseng^11^,^12^,^13^. Furthermore, both Asian ginseng and American ginseng have anti-cancer effects by modulating signaling pathways associated with inflammation, oxidative stress, angiogenesis, metastasis, and stem/progenitor-like properties of cancer cells^14^.

Over the past decades, most of the pharmacology researches on Asian ginseng and American ginseng are independent conducted, few is carried out simultaneously. With the character of complex composition, ginseng usually acts on multireceptor systems or non-target impacts such as intestinal microbiota^15^. Thus, we need seek for integrative approach to deepen pharmacology mechanism. As an important part of systems biology, metabolomics characterized by holistic perspective which is consistent with the integral thinking of traditional Chinese medicine (TCM) and the comprehensive actions of TCM has been applied to pharmaceutical industry, drug toxicity and clinical diagnosis^16^,^17^,^18^. Modern pharmacological tools provide a favorable opportunity to accelerate research on mechanism of action of herbs, especially a systems-biology-based pharmacological study of TCM^19^. In the period of systems biology, metabolomics abridge TCM with molecular pharmacology to an in-depth understanding of molecular mechanism and signal transduction pathway^20^.

In recent metabolomics study, most of which have focused on chemical markers and quality control between Asian ginseng and American ginseng^21^,^22^,^23^,^24^. This work provides new insight into different metabolome disturbance in urine along with a four-week administration of Asian ginseng and American ginseng coupling the GC-MS platform which presents unique strengths of high sensitivity, reproducibility and available spectral libraries^25^. Because urine contains most of the body’s metabolic end products revealing the state of the organism and its noninvasive feature, urinary metabolic profiling has been a favorable technique and has been adopted in this protocol^26^.

Both Asian ginseng and American ginseng are recognized as tonic herbs and used as dietary supplement worldwide due to its regulation and promotion on body. In our study, we target ginsengs in their nourishing functions and aim to further guide for ginseng daily use in different physical crowd. Also, this study also offers new insight into TCM properties and their traditional application.

## Results

### Metabolic changes in response to Asian ginseng and American ginseng treated mice

**Figure 1:**
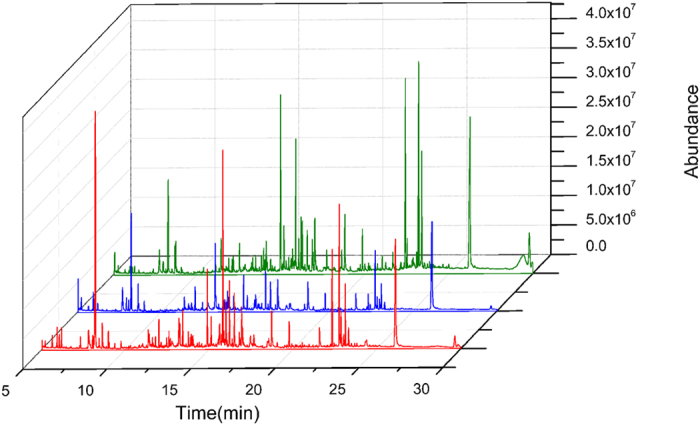
Typical total ion current (TIC) chromatograms of the three groups obtained from GC-MS analysis. Red represents CON group, blue represents ASG group, green represents AMG group.

**Figure 2:**
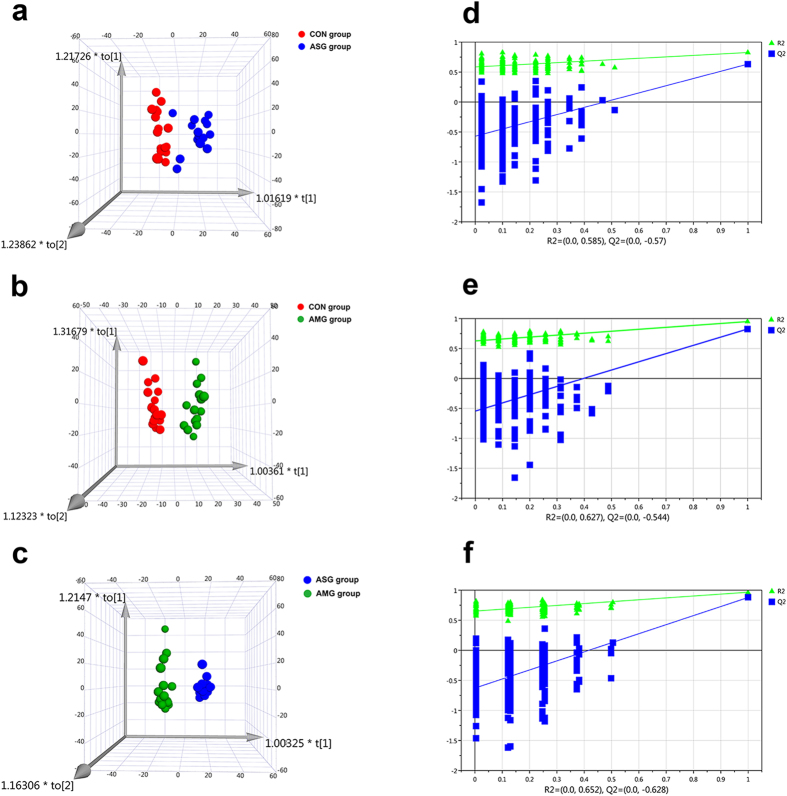
OPLS-DA score plots for discriminating the urine metabolome from control group and treatment group and corresponding permutation test obtained from GC-MS. (**a**) CON *vs* ASG group, (**b**) CON *vs* AMG group, (**c**) ASG *vs* AMG group. Chance permutation at 200 times was used for the discrimination between (**d**) CON *vs* ASG, (**e**) CON *vs* AMG, and (**f**) ASG *vs* AMG.

GC-MS was a powerful tool for metabolomics research and it extracted a total of 1797 metabolite peaks in this study. The representative total ion current (TIC) chromatograms obtained from Control group (CON group), Asian ginseng group (ASG group) and American ginseng group (AMG group) were shown in Fig. 1. Although subtle differences between control and treatment groups could be observed from chromatograms, it was still inadequate to clarify definite differentiation exists among control, Asian ginseng and American ginseng administration group. Therefore, intergroup differences were represented by constructing orthogonal partial least squares discriminant analysis (OPLS-DA) model in Fig. 2. It is obvious that clear separation was observed between CON group and ASG group (R2Y (cum) = 0.826, Q2 (cum) = 0.633, Fig. 2a) as well as CON group and AMG group (R2Y (cum) = 0.951, Q2 (cum) = 0.827, Fig. 2b), which indicated urinary metabolic pattern has changed in response to Asian ginseng and American ginseng treated mice. Moreover, a well-separated tendency from Asian ginseng and American ginseng individuals was similarly displayed, suggesting that a different metabolic phenotype existed between Asian ginseng and American ginseng (Fig. 2c). The parameters R2Y (cum) and Q2 (cum) value of 0.966 and 0.879 were considered to be a good fitness and predictability of the constructed OPLS-DA model, respectively (Fig. 2c). Metabolomics usually covered megavariate dataset which meant that the total number of variables is much larger than the number of observations, may leading a chance correlation^26^,^27^. To validate the model and avoid overfitting, permutation test (n = 200) was necessary and presented. As shown in Fig. 2d–f, the GC-MS analytical platform has excellent performance in stability and repeatability, and could be exploited in subsequently metabolomics research.

**Figure 3:**
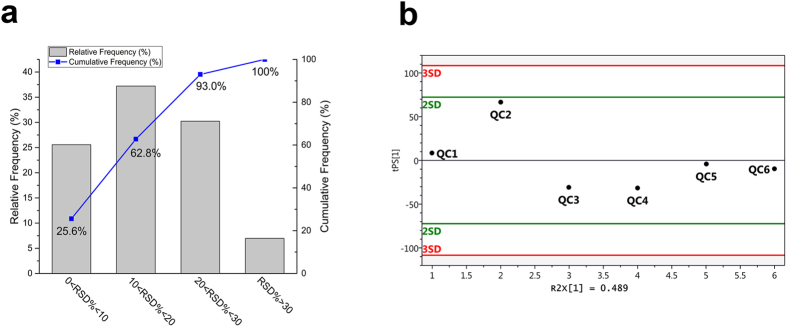
Analytical variation determined by quality control (QC) samples performance. (**a**) Distribution of RSD% for differential metabolites’ peak area in 6 QC samples, (**b**) PCA first component score plot for QC samples.

The reproducibility of analysis was assessed by using QC samples. The relative standard deviations (RSD%) were calculated differential metabolites concentrations in the QC samples. As is shown in Fig. 3, 93% of differential metabolites have a RSD% less than 30% and QC samples behaved stable for the duration of the run. Based on these results, the high quality data was sufficient to warrant further statistical analysis of the results to detect biomarkers^28^.

### Identification of Asian ginseng-responsive and American ginseng-responsive differential metabolites

Differential metabolites contributing to the separation were identified using variable importance in the projection (VIP) value and p value. In general, a threshold of VIP >1 was considered as the relevant metabolites for interpreting the discrimination and Wilcoxon-Mann U test p value set to 0.05 (p < 0.05) was believed to a significant difference. Based on above strategy, a total of 21 and 34 discriminating metabolites resulting from Asian ginseng and American ginseng treatment groups were respectively selected (Tables 1 and 2). The significant changed metabolites were distributed as organic acids, amino acids and sugar alcohols, and 12 common metabolites were altered after Asian ginseng and American ginseng exposure.

**Figure 4:**
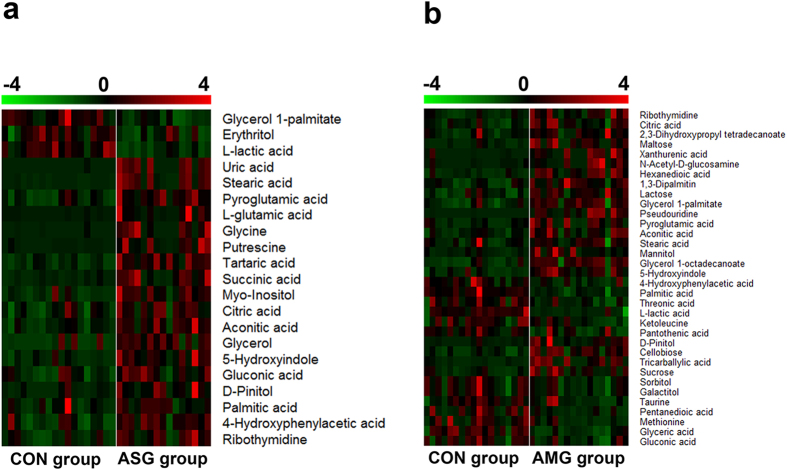
Heatmaps visulization of the differential metabolites responding to Asian ginseng and American ginseng. (**a**) ASG group compared to CON group, (**b**) AMG group compared to CON group. The colors from green to red indicates the increasing expression of metabolites.

**Figure 5:**
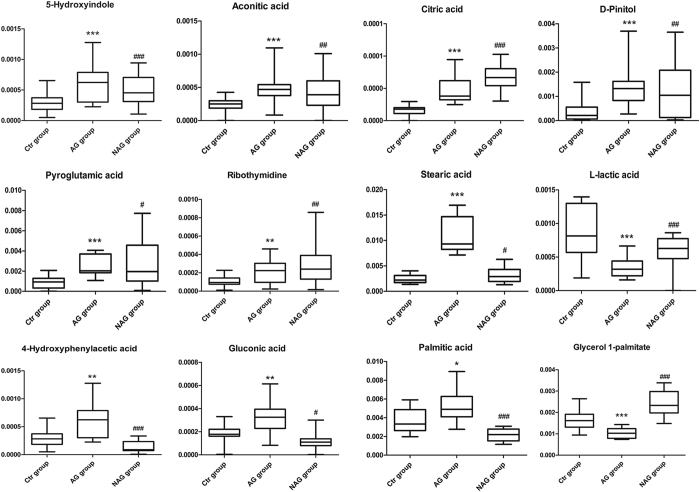
Box plots showing the levels of 12 common differential metabolites in the CON group, ASG group and AMG group. *p < 0.05, **p < 0.01, ***p < 0.001 for ASG group *vs* CON group; ^#^p < 0.05, ^##^p < 0.01, ^###^p < 0.001 for AMG group *vs* CON group.

To intuitively inspect the tendency of the variation of the metabolite concentrations between non-treated control group and treatment groups, heat maps were produced according to the relative quantities of each marker. As shown in Fig. 4, compared to CON group, the content of 18 metabolites increased and 3 metabolites decreased in ASG group, while 21 metabolites enhanced and 13 metabolites reduced in AMG group. Among 12 common altered metabolites, the excretion of lactic acid was both declined under the two treatments, whereas coordinated expanded in the content of 7 metabolites including pyroglutamic acid, 5-hydroxyindole, aconitic acid, citric acid, D-pinitol, stearic acid and ribothymidine. In addition, 4-hydroxyphenylacetic acid, gluconic acid and palmitic acid displayed a converse trend that enlarged in response to Asian ginseng and fell in response to American ginseng while glycerol 1-palmitate had an opposite tendency (Fig. 5).

### Metabolic pathway analysis of Asian ginseng and American ginseng

**Figure 6:**
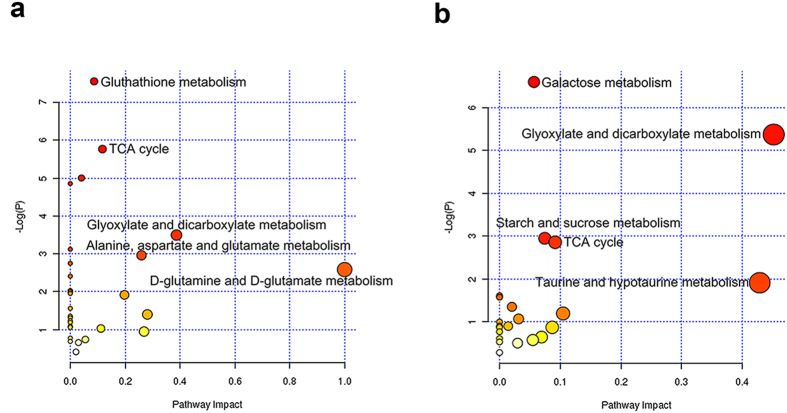
Pathway mapping based on the differential metabolites. (**a**) ASG group versus CON group, (**b**) AMG group versus CON group.

**Figure 7:**
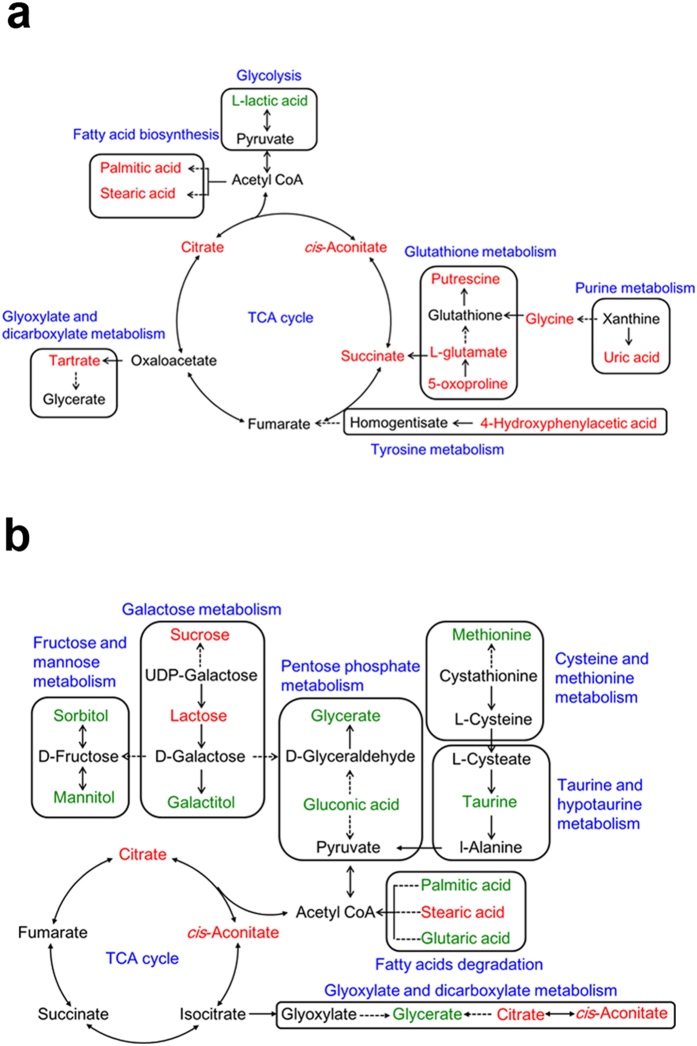
Metabolic pathway networks of significant different metabolites in response to Asian ginseng (**a**) and American ginseng (**b**). The level of metabolites compared to that of control group is marked in red (up-regulated) and green (down-regulated), respectively. The black color represents the undetected metabolites. The words in blue besides the pane indicate the related pathways.

Metabolic pathway analysis was further studied on the basis of the important metabolites listed in Tables 1 and 2. MetaboAnalyst (www. metaboanalyst. ca) is a web-based server supporting pathway analysis which integrated enrichment analysis and pathway topology analysis to reveal the significant relevant pathways influenced by Asian ginseng and American ginseng administration, respectively. Figure 6a showed that elevated D-glutamine and D-glutamate metabolism was thought to be involved in the most relevant pathway influenced by Asian ginseng with the impact value of 1.0. In addition, another four pathways including glutathione metabolism, TCA cycle, glyoxylate and dicarboxylate metabolism as well as alanine, aspartate and glutamate metabolism were promoted responding to Asian ginseng intervention compared with control group. Meanwhile, it can be seen that American ginseng administration upregulated two pathways related to glyoxylate and dicarboxylate metabolism pathway and TCA cycle whereas down-regulated three pathways such as taurine and hypotaurine metabolism pathway, galactose metabolism pathway as well as starch and sucrose metabolism pathway which filtered out to the important metabolic pathways (Fig. 6b). A metabolic network was constructed by searching the KEGG pathway database (Fig. 7).

## Discussion

In this study, we describe urinary metabolic footprints change responding to Asian ginseng and American ginseng intragastric administration using GC-MS techniques combined with multivariate analysis. Urine is a complex matrix mixing with lots of end products of metabolites (including pharmacy) such as fatty acids, steroids, amino acids and esters. However, some of compounds cannot be detected by GC-MS owing to its nature of non-volatile and polar. Chemical derivatization serves functions of reducing the polarity, increasing the thermal stability and volatility of the analytes^25^. Resultantly, we adopt a two-step derivatization procedure in which the residue is subjected to oximation using methoxyamine hydrochloride followed by trimethylsilyl derivatization using *N,O-Bis(trimethylsilyl)trifluoroacetamide* (BSTFA) with 1% *trimethylchlorosilane* (TMCS).

L-glutamic acid is believed to be a predominant position in 21 altered metabolites in response to Asian ginseng, the level of which elevated 20 times compared with that of control group. Furthermore, glutamate-centered metabolism (D-glutamine and D-glutamate metabolism, glutathione metabolism, alanine, aspartate and glutamate metabolism) was considered to be the most relevant pathways influenced by Asian ginseng. As the principal excitatory neurotransmitter in brain, glutamic acid plays a crucial role in functions of learning and memory^29^. It can be suggested that Asian ginseng improve cognitive performance via modulate glutamate release in agreement with the previous study^30^. All Asian ginseng-regulated metabolites involved in glutathione metabolism were increased which shows protection on tissues from toxic molecules produced by oxidative stress^31^. These include L-glutamic acid (20.3 fold enrichment), glycine (29.6 fold enrichment), pyroglutamic acid (2.9 fold enrichment) and putrescine (10.3 fold enrichment).

A panel of altered saccharide metabolites including sorbitol, galactitol, sucrose, lactose and maltose which is responsible for galactose metabolism as well as starch and sucrose metabolism pathway presents a strong link to glycometabolism in AMG group. A relative low level of monosaccharides such as sorbitol and galactitol as well as a high level of disaccharides including sucrose, lactose and maltose points out that a biotransformation from monosaccharide to disaccharide has been enhanced. These results agree with the hypoglycemic activity of American ginseng published previously^13^. In addition, decreasing content of taurine in AMG group indicates the disturbance of taurine and hypotaurine metabolism. Taurine is a sulfur amino acid that is highly abundant in excitable tissues, especially in brain and heart. It has been proven that the protective effects of taurine appear to involved antioxidant, osmoregulatory and ion regulatory activities^32^. An inconspicuous descending tendency found in our results implies that American ginseng is likely to enhance taurine utilization followed by reduction of excretion under physiological conditions.

**Figure 8:**
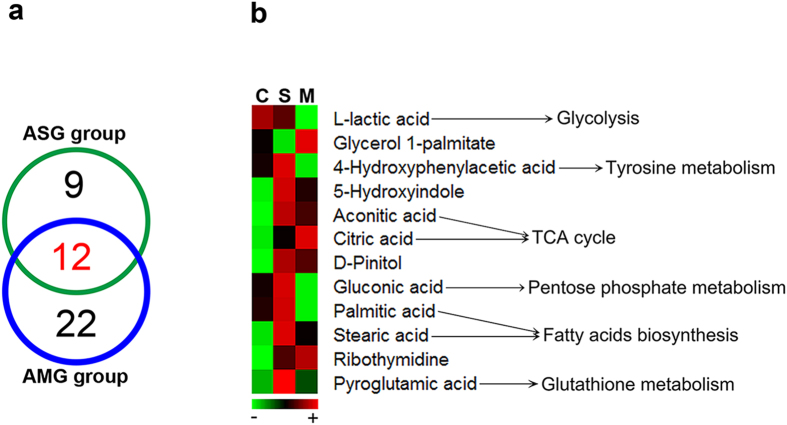
A panel of 12 common differential metabolites from ASG and AMG group and their related pathways. (**a**) Venn diagram showing the number of metabolites exhibiting a significant difference from ASG and AMG group, (**b**) Heatmaps of 12 common differential metabolites contructed from average normalized peak areas of three groups and their related metabolic pathways. C: CON group, S: ASG group, M: AMG group.

Moreover, 12 common differential metabolites from ASG and AMG group perform a closed connection to energy expenditure (Fig. 8). The decreased level of lactic acid produced from aerobic glycolysis (Warburg effect) is attributed to anticancer action of ginseng^33^,^34^. These observations are similar with the data showed here regarding low urinary lactic acid in ASG and AMG group. D-pinitol, an active antidiabetic principle isolated from natural herbs^35^, was observed at a high level in Asian ginseng and American ginseng group, which might lead to the improved glycaemic control. In epidemiologic and clinical studies, stearic acid was found to be associated with lowered LDL cholesterol in comparison with other saturated fatty acids^36^. A significant enrichment in stearic acid was found in Asian ginseng treatment group, implying Asian ginseng probably attenuate cardiovascular disease risk resulted from elevated content of stearic acid. Different trends in gluconic acid lead to the variation level of NADPH which is generated from pentose phosphate pathway and NADPH is employed to fatty acids biosynthesis, which corresponds with the level of palmitic acid influenced by Asian ginseng and American ginseng.

Intriguingly, both Asian ginseng and American ginseng elevate the level of aconitic acid and citric acid which belongs to glyoxylate and dicarboxylate metabolism pathway as well as TCA cycle highly associated with energy metabolism. Furthermore, it is well-established that Asian ginseng has been widely used for replenishing qi, so as to American ginseng. Such restoration of potency may partially attribute to the improvement of energy metabolism consistent with the experiment conducted in deficiency of vital energy rat^37^. 4-hydroxyphenylacetic acid responsible for tyrosine metabolism is enrichment in ASG group, however, appears a lower strength in AMG group. The previous literature has showed that spleen-deficiency patients are characterized by disorder of tyrosine metabolism^38^, and it is general knowledge that both Asian ginseng and American ginseng have been used for invigorating the spleen, but present diversified ability. In the attributive channel theory of Chinese herbal medicine, both Asian ginseng and American ginseng are attributable to the kidney. The improvement in renal function may due to increased urinary content of pyroglutamic acid identified from ASG and AMG group. Also, metabolomics study has uncovered that a clear decrease in pyroglutamic acid in urine of chronic kidney disease (CKD) patients and a higher level of pyroglutamic acid is significantly related to lower risk of incident CKD^39^,^40^.

In summary, our present work do a research on comparison of physiological function between Asian ginseng and American ginseng with a systematic metabolomics method. Clearly, a distinct metabolic pattern between Asian ginseng and American ginseng is discovered by OPLS-DA with a panel of urinary characteristic metabolites. The important pathway identified from Asian ginseng and American ginseng may be useful for further mechanism analysis and can also help to explore new therapeutic effects or action targets so as to broad application of Asian ginseng and American ginseng. It is notable that several fatty acids and amino acids which are primary metabolites in Asian ginseng and American ginseng^41^,^42^,^43^ were highlighted as the differential metabolites in response to the treatments of two ginsengs, the effect of exogenous metabolites should take into consideration in future study. However, there still are some limitations on this study. First, a normal animal model instead of disease model adopted in this paper might not be in the direction of target action mechanism. Second, all metabolites are only detected by a single technique of GC-MS. Thus, further analysis combined with LC-MS should be considerable.

## Materials and Methods

### Chemicals

Chromatographic grade methanol was from Hanbang Tech Co. (Jiangsu, China). Urease (Type III), methoxyamine hydrochloride, N,O-Bis(trimethylsilyl)trifluoroacetamide (BSTFA) with 1% trimethylchlorosilane (TMCS) and all standard compounds were purchased from Sigma-Aldrich. Ultrapure water was prepared using a Milli-Q purification system. Six-year-old Asian ginseng (*Panax ginseng*) was provided by Infinitus (China) Company Ltd. American ginseng (Panax quinquefolius) was obtained from BaiXin Pharmacy (Nanjing, China). Asian Ginseng and American ginseng were pulverized through a 100-mesh sieve and suspended in ultrapure water for animal experiments.

### Animal protocols

All procedures were conducted in accordance with Administrative Measures of Experimental Animals in Jiangsu Province, and the experimental protocols were approved by the Animal Ethics Committee of China Pharmaceutical University. Fifty-four male ICR mice (18–22 g) were purchased from the Experimental Animal Center of Yangzhou University (Yangzhou, China) and housed under controlled environmental conditions (23–27 °C and 12 h light/dark cycle) with free access to standard diet and water. After one week adaptive feeding, the mice were randomly divided into three groups: CON group (n = 18), ASG group (n = 18) and AMG group (n = 18). The treatment groups were intragastrically administrated with Asian ginseng solution (2 g/kg) and American ginseng solution (2 g/kg) once a day, respectively. And the control group was dosed with equivalent volumes of ultrapure water. 24 h urine was collected from all mice in individual metabolic cages after 30 days administration. All urine samples were immediately centrifuged at 3000 rpm for 10 min and the supernatants were stored at −80 °C until analysis.

### Sample preparation and analysis

Urine samples were prepared following previous published protocol with minor modification^26^. An aliquot of 50 μl urine sample was mixed with 120 μl urease (10 mg/ml, 37 °C, 60 min) to remove high content of urea. 400 μl methanol was used to precipitate protein followed by vortex mixing for 5 min before centrifuged at 10000 g for 10 min at 4 °C. A 300 μl aliquot of supernatant was transferred to a clean Eppendorf tube and was dried under a gentle stream of nitrogen gas. The residue was derivatized by addition of 40 μl methoxyamine hydrochloride (15 mg/ml in pyridine) at 60 °C for 2 h. To each sample add 60 μl BSTFA (1% TMCS) and heat the mixture at 70 °C (1200 rpm) for 60 min. Allow the derivative to cool and centrifuge at 13000 rpm for 10 min prior to GC-MS analysis. Quality control (QC) samples were prepared by pooling aliquots of all the urine samples and were processed with the same procedure as that for the experiment samples.

Each 2 μl aliquot of derivatized sample was injected in 30:1 split ratio into an Agilent 7890B/5977A gas chromatography-mass spectrometry (GC-MS) equipped with HP-5MS capillary column (30 m × 0.25 mm × 0.25 μm, Agilent J & W Scientific, USA). Helium was used as the carrier gas with a constant flow rate of 1 ml/min. The temperature program was as follows: the initial temperature was 80 °C hold for 2 min, elevated to 300 °C at a rate of 10 °C/min and was maintained for 6 min. The temperature of the injector, transfer line and ion source was set to 250, 280 and 230 °C, respectively. The mass range (50–600 m/z) in a full-scan mode for electron impact ionization (70 eV) was applied. The solvent delay time was set to 5 min.

### Data processing and statistical analysis

Raw GC-MS data were exported to mzData format by MassHunter Workstation Software (Version B.06.00, Agilent Technologies) and subsequently sent to XCMS package under the R Project. The pretreatment process included novel nonlinear retention time alignment, baseline filtration, peak identification, matching and integration. The resulting data matrix which consists of variables, sample code and peak area was further processed using Microsoft Excel 2010. Total chromatographic area normalization was applied to reduce the deviation originates from discrepant urine concentration between each sample. Finally, the normalized dataset was imported into SIMCA 14 (Umetrics, Sweden) for multivariate statistically analysis and SPSS (Version 19) for univariate data analysis.

To verify separation trends between different groups, supervised orthogonal partial least squares discriminant analysis (OPLS-DA) was used. On the basis of variable importance in the projection (VIP) values greater than 1.0 obtained from OPLS-DA model and p values less than 0.05 acquired from Wilcoxon-Mann U test, a set of discriminating metabolites were determined. All metabolites were identified by comparing mass fragments with the standard mass spectra on the commercial database NIST with a similarity of more than 70%.

## Additional Information

**How to cite this article**: Yang, L. *et al*. Distinct urine metabolome after Asian ginseng and American ginseng intervention based on GC-MS metabolomics approach. *Sci. Rep.* **6**, 39045; doi: 10.1038/srep39045 (2016).

**Publisher's note:** Springer Nature remains neutral with regard to jurisdictional claims in published maps and institutional affiliations.

## Figures and Tables

**Table 1:** Differential metabolites in response to Asian ginseng treatment.

No.	RT (min)	Compound	Formula	VIP value^a^	p value^b^	FC^c^	Trend
1	21.70	Glycerol 1-palmitate*	C_19_H_38_O_4_	1.646	1.11E-04	0.631	Down
2	6.42	Tartaric acid	C_4_H_6_O_6_	1.607	5.03E-06	4.130	Up
3	11.49	Pyroglutamic acid*	C_5_H_7_NO_3_	1.604	3.33E-05	2.945	Up
4	18.78	Stearic acid*	C_18_H_36_O_2_	1.537	1.34E-04	4.558	Up
5	17.76	Uric acid*	C_5_H_4_N_4_O_3_	1.475	3.86E-04	40.648	Up
6	5.15	L-Lactic acid*	C_3_H_6_O_3_	1.471	6.66E-06	0.352	Down
7	14.10	Aconitic acid*	C_6_H_6_O_6_	1.370	9.18E-05	2.499	Up
8	8.24	Glycerol	C_3_H_8_O_3_	1.336	2.37E-03	2.815	Up
9	13.70	5-Hydroxyindole	C_8_H_7_NO	1.305	1.72E-05	2.672	Up
10	21.03	Ribothymidine*	C_10_H_14_N_2_O_6_	1.285	1.56E-03	2.426	Up
11	14.01	Putrescine*	C_4_H_12_N_2_	1.256	1.37E-05	10.298	Up
12	17.70	Myo-Inositol*	C_6_H_12_O_6_	1.221	1.61E-04	2.433	Up
13	11.43	Erythritol*	C_4_H_10_O_4_	1.221	1.47E-02	0.684	Down
14	8.69	Glycine*	C_2_H_5_NO_2_	1.191	2.75E-04	29.617	Up
15	15.19	D-Pinitol	C_7_H_14_O_6_	1.166	1.00E-03	4.144	Up
16	8.71	Succinic acid	C_4_H_6_O_4_	1.141	7.47E-03	4.862	Up
17	12.80	4-Hydroxyphenylacetic acid*	C_8_H_8_O_3_	1.113	2.37E-03	1.608	Up
18	17.00	Palmitic acid*	C_16_H_32_O_2_	1.047	1.82E-02	1.973	Up
19	12.65	L-Glutamic acid*	C_5_H_9_NO_4_	1.047	3.86E-04	20.301	Up
20	14.961	Citric acid*	C_6_H_8_O_7_	1.031	5.19E-03	3.083	Up
21	16.54	Gluconic acid	C_6_H_12_O_7_	1.015	8.40E-03	1.855	Up

^a^Variable importance in the projection (VIP) was obtained from OPLS-DA with a threshold of 1.0.

^b^p value was calculated from Wilcoxon-Mann U test.

^c^Fold change (FC) was calculated from the arithmetic mean values between ASG and CON group. The metabolites marked with “*” were structurally identified by reference standards.

**Table 2:** Differential metabolites in response to American ginseng treatment.

No.	RT (min)	Compound	Formula	VIP value^a^	p value^b^	FC^c^	Trend
1	17.00	Palmitic acid*	C_16_H_32_O_2_	1.633	8.82E-10	0.687	Down
2	5.13	L-Lactic acid*	C_3_H_6_O_3_	1.541	2.43E-05	0.682	Down
3	9.05	Glyceric acid	C_3_H_6_O_4_	1.480	2.30E-05	0.335	Down
4	19.83	Pseudouridine	C_9_H_12_N_2_O_6_	1.349	4.03E-04	21.931	Up
5	21.03	Ribothymidine*	C_10_H_14_N_2_O_6_	1.320	3.82E-03	2.893	Up
6	23.07	Glycerol 1-octadecanoate*	C_21_H_42_O_4_	1.298	1.03E-05	2.176	Up
7	23.18	Cellobiose*	C_12_H_22_O_11_	1.296	1.34E-04	2.898	Up
8	13.90	Tricarballylic acid	C_6_H_8_O_6_	1.289	8.34E-03	8.748	Up
9	22.38	Sucrose*	C_12_H_22_O_11_	1.288	8.34E-03	2.854	Up
10	13.41	3-Hydroxyadipic acid	C_6_H_10_O_5_	1.286	4.67E-04	3.432	Up
11	21.74	1,3-Dipalmitin	C_35_H_68_O_5_	1.275	4.67E-04	1.979	Up
12	13.70	5-Hydroxyindole	C_8_H_7_NO	1.264	2.18E-04	2.043	Up
13	23.37	Maltose*	C_12_H_22_O_11_	1.262	5.39E-03	4.248	Up
14	12.05	Threonic acid	C_4_H_8_O_5_	1.260	2.16E-03	0.530	Down
15	16.27	Sorbitol*	C_6_H_14_O_6_	1.250	4.87E-02	0.691	Down
16	16.31	Galactitol*	C_6_H_14_O_6_	1.246	4.13E-02	0.693	Down
17	15.18	D-Pinitol	C_7_H_14_O_6_	1.181	4.82E-03	3.231	Up
18	16.55	Gluconic acid	C_6_H_12_O_7_	1.176	4.13E-02	0.688	Down
19	13.26	Taurine*	C_2_H_7_NO_3_S	1.170	2.24E-02	0.789	Down
20	16.66	Pantothenic acid*	C_9_H_17_NO_5_	1.162	1.36E-02	0.661	Down
21	16.25	Mannitol*	C_6_H_14_O_6_	1.159	5.39E-03	3.116	Up
22	19.13	Xanthurenic acid*	C_10_H_7_NO_4_	1.156	5.70E-03	7.300	Up
23	21.66	Glycerol 1-palmitate*	C_19_H_38_O_4_	1.156	2.98E-04	1.467	Up
24	12.87	4-Hydroxyphenylacetic acid*	C_8_H_8_O_3_	1.155	6.03E-03	0.437	Down
25	12.15	Glutaric acid	C_5_H_8_O_4_	1.125	3.40E-03	0.349	Down
26	14.98	Citric acid*	C_6_H_8_O_7_	1.120	4.28E-04	4.327	Up
27	14.10	Aconitic acid*	C_6_H_6_O_6_	1.093	2.48E-03	2.233	Up
28	11.53	Pyroglutamic acid*	C_5_H_7_NO_3_	1.087	2.45E-02	1.372	Up
29	7.34	Ketoleucine*	C_6_H_10_O_3_	1.081	4.07E-02	0.643	Down
30	20.21	1-Myristoyl-glycerol	C_17_H_34_O_4_	1.076	3.02E-03	1.207	Up
31	18.79	Stearic acid*	C_18_H_36_O_2_	1.066	2.24E-02	1.076	Up
32	23.15	Lactose*	C_12_H_22_O_11_	1.061	7.20E-04	1.641	Up
33	10.01	Methionine*	C_5_H_11_NO_2_S	1.049	2.45E-02	0.366	Down
34	17.67	N-Acetyl-D-glucosamine	C_8_H_15_NO_6_	1.009	1.39E-02	4.973	Up

^a^Variable importance in the projection (VIP) was obtained from OPLS-DA with a threshold of 1.0.

^b^p value was calculated from Wilcoxon-Mann U test.

^c^Fold change (FC) was calculated from the arithmetic mean values between AMG and CON group. The metabolites marked with “*” were structurally identified by reference standards.
